# Scent in Motion: On the Multiple Uses of Ambient Scent in the Context of Passenger Transport

**DOI:** 10.3389/fpsyg.2021.702517

**Published:** 2021-07-12

**Authors:** Charles Spence

**Affiliations:** Head of the Crossmodal Research Laboratory, University of Oxford, Oxford, United Kingdom

**Keywords:** transport, malodour, scent, functional scents, signature scents, scent marketing

## Abstract

There is undoubtedly growing interest in the role of scent in the design of multisensory experiences. However, to date, the majority of the research has focused on its use in the (static) built environment. As highlighted by this narrative review, somewhat different challenges and opportunities arise just as soon as one starts to consider olfaction in the case of transportation–what might be called “scent in motion.” For instance, levels of anxiety/stress while traveling are often higher (especially in the case of air travel), while, at the same time, the passenger's personal space is frequently compromised. Four key functional roles for scent in the context of passenger transportation are outlined. They include the masking of malodour, the introduction of branded signature scents, short-term olfactory marketing interventions, and the functional use of scent to enhance the experience of travel. In the latter case, one might consider the use of scent to help reduce the stress/anxiety amongst airplane passengers or to give the impression of cleanliness. Meanwhile, in the case of driving, scents have been suggested as an inoffensive means of alerting/relaxing the driver and may also help tackle the problem of motion sickness. The specific challenges associated with scent in motion are reviewed and a number of future opportunities highlighted.

## Introduction

Scent plays an important role in our experience of the built environment (see Spence, 2020c, for a recent review). This should come as little surprise when it is realized that the world's majority urban population spends an estimated 90–95% of their time indoors (Ott and Roberts, 1998; Klepeis et al., 2001; Wargocki, 2001; Velux YouGov Report, 2018)^1^. In this review, I will consider the role played by ambient scent in passenger transportation–what might be considered “scent in motion,” though, as we will see later, sometimes it will be more a case of “stench in motion” (Robinson, 2016). To give some context, North Americans spend an average of an hour a day behind the wheel (https://www.volpe.dot.gov/news/how-much-time-do-americans-spend-behind-wheel), be it the daily commute or the school run (see also Chapin, 1974). This represents a not inconsiderable source of stress for many people, especially when their travel is, for whatever reason, impeded (Novaco et al., 1990). Indeed, commuting/travel is one of the few aspects of our daily lives that has not gotten any faster as the decades have gone by Colvile (2017). At present, average speeds in the world's growing number of megacities are around 15 km/h (Nyirinkindi and Paris, 2020), and that figure is predicted to drop down to 3 km/h by 2030.

Regardless of the sector (be it healthcare, retail, etc.), increasing competition in the marketplace typically leads to a greater focus on the experience. In the case of passenger transportation, the shift in focus is from the final destination and the duration/cost of the trip to a greater emphasis on the experiential qualities of the journey itself (as probed by the all too frequent request for feedback along the lines of “How was the experience?”). The much anticipated arrival of space tourism further promises to elevate transportation to an entirely new level, at least for the lucky few who can afford to partake in this admittedly niche activity (see Henderson and Tsui, 2019). Indeed, transportation is an especially dynamic sector currently, with radical challenges to conventional business models an increasingly common occurrence—-think here only of the disruption offered by the likes of Neutonomy, Uber, and Tesla (see Smart, 2017; Woodward et al., 2017; Spence, 2021a; though see also Diao et al., 2021; Liberatore, 2021, on the downside of rideshare apps, otherwise referred to as Transportation Network Companies (TNCs), such as Uber and Lyft). What is more, the introduction of flying taxis may be closer than many people think (The Local, 2019; Pasztor and Tangel, 2020; see also Murphy, 2021). All that before we get to Richard Branson's Hyperloop Propulsion Pods, with matching signature scent (see Giacobbe, 2021). Given the highly-dynamic and competitive nature of the transportation market, there is a growing opportunity to enhance/differentiate the multisensory travel experience for passengers via design that targets the sense of smell.

That said, when thinking about the multisensory experience of travel, it is important to consider how our experience, especially of public transport, typically involves not only the time spent on the move but often requires the passengers to transit through, and often wait in, stations (see Schivelbusch, 2014, on the early history of the railway station), airports (Wattanacharoensil, 2019), bus/tram stops, etc. These spaces are, properly considered, therefore a part of the experience of travel too (cf. Goetz, 2019), though equally also fit within any discussion of scent in the built environment. Indeed, given the importance of primacy/recent effects to human memory, they may actually play a disproportionately important role in the passengers' memory of their travel experiences (see Carbone and Haeckel, 1994; Berry et al., 2002; Carbone, 2004; LaTour and Carbone, 2014, on the notion of “Sticktion”). Hence, in the narrative review that follows, I will look at the role of scent in all stages of the journey and across a wide range of different forms of travel. As we will see in a moment, though, while many of the same issues and opportunities present themselves in the case of scent in the built environment (static) and in the case of dynamic scent (i.e., transport), there are also a number of salient differences. It should also be noted that, as has just been mentioned, the distinction between scent in motion and scent's use in the built (i.e., static) environment is by no means always clear-cut, with stations, airports, etc. fitting naturally into both categories.

### Air Pollution

The topic of air pollution is also very relevant to the themes of this review inasmuch as many forms of transportation are associated with high levels of air pollution, some of which are detected consciously by those who are themselves traveling, or else by others who may be in the vicinity. Indeed, levels of air pollution in and around different forms of transportation (i.e., both for those who are traveling and those who find themselves nearby) tend to be much higher than is commonly the case when considering the olfactory atmosphere of the built environment. For instance, just take Oxford Street in London, often mentioned as one of the most polluted streets in the world in terms of traffic-related air pollution (cf. Popovich et al., 2019). The levels of nitrogen dioxide (NO_2_) recorded on this world-famous shopping street are dangerously (not to mention illegally) high more than 80% of the time.

The deleterious effects on respiratory health of such transport-related pollution are well-documented (WHO Technical Report, 2013; van Veldhoven et al., 2019). Increased levels of air pollution may result in increased road rage amongst drivers. Consistent with such a view, almost 50 years ago, researchers in California had already highlighted how the number of road traffic accidents in Los Angeles correlated with the level of air pollution (Ury et al., 1972)^2^. Relevant here, therefore, is recent research reported by Dmitrenko et al. (2020) showing how pleasant scents can be used to promote safer driving, better mood, and improved well-being in angry drivers.

The air that people breathe in (in particular, the particulate matter) when traveling on underground transportation can be pretty toxic (cf. Gouveia and Maisonet, 2005; Paton, 2019; Radnedge, 2019), with a trip on the London Underground, the dirtiest in the world, exposing passengers to dangerously high levels of particulate matter. The levels of toxic airborne pollutants in the atmosphere in the London Underground, are currently almost 20 times higher than recommended by the World Health Organization (WHO). Given such shocking figures, one might think that adding a pleasant scent is probably the last thing that commuters should be worrying about. As another example of the olfactory pollution that was once associated with travel, one need only think back to the days when a flimsy cloth curtain was all that separated the smoking section at the back of the airplane from the rest of the non-smoking passengers. While a separate non-smoking section has been mandated in planes in the US since 1973, smoking was only banned completely on flights to/from or within the US in 2000, while the ban only came in much later in China; c. 2016; (Pallini, 2020). Given the well-established dangers of passive smoking, this would also have to constitute a pretty polluted olfactory environment (Crawford and Holcomb, 1991; Repace, 2004). Furthermore, one of the arguments put forward by those who are against airport expansion relates to the likely increase in air pollution experienced by those living in surrounding neighborhoods (e.g., see Anon, 2021b).

Fortunately, however, most of us typically spend much less time exposed to such pollutants (which may, or may not, be perceptible consciously), meaning that any deleterious effects on our health and well-being are, if not mitigated entirely, at least reduced significantly as compared to the volatile organic compounds (VOCs) that are typically found in poorly-ventilated home or office environments where we spend so much more of our time (see Spence, 2002, 2021a; though see also Watson, 2021). Here, for instance, one might wonder whether there is an equivalent to the sick-building syndrome (SBS; Spence, 2002, 2020c; Love, 2018) that was sometimes reported half a century ago—-something of the sort of sick transport syndrome (STS; a quick online search reveals the existence of Sick Yacht Syndrome, SYS; https://www.rgf.com/article/sick-yacht-syndrome/)?^3^ There is, though, a separate question about exposure to air pollution amongst those who work in the transportation/travel industries (Repace, 2004; Bloomberg, 2017). Finally here, it is worth highlighting how the emergence of the Covid-19 pandemic, has bought into sharp focus the potentially infectious atmosphere while traveling on public transport (e.g., see Anon, 2021a; Bunyan, 2021)–and see Luke et al. (2020) for the crucial role that transportation played in facilitating the spread of the Spanish Flu a century earlier.

### Anxiety

One of the other noticeable differences between scent in the built environment (see Spence, 2020a, Spence, 2020b) and scent in motion (e.g., in transportation) relates to the fact that for many of us, travel (especially air travel) is a major cause of anxiety. Historically, anxiety associated with transportation has often been triggered by the introduction of new, often much faster, forms of travel, such as, for example, the train (i.e., for those who were used to traveling by horse-drawn carriage or stagecoach; see Schivelbusch, 2014). Indeed, the palpable concerns of many early rail passengers (and a number of eminent doctors/medics), was eloquently brought out in Schivelbusch's 2014, *The Railway Journey*. Similarly, when the first public elevator was introduced in 1857 in the US (described in the initial patent as a “vertical railway”; Prisco, 2019), at the Manhattan Department store E.W. Haughtwhat & Company in New York it was not a success, closing 3 years later (Bernard, 2014; Prisco, 2019). In this case, it may well have been the confined nature of the space (possibly triggering claustrophobia), not to mention the hidden nature of the underlying operations (mechanisms), that made the public nervous (e.g., Bernard, 2014; cf. Schivelbusch, 2014). The anxiety that this once novel form of transport gave rise to initially was eventually ameliorated by the introduction of mirrored surfaces (to show the passengers who else is in the lift with them, while also making the space look bigger) and the so-called busboy (whose presence continued long after it was strictly needed). Elevator music may also have been used to help calm those who felt anxious (see Friedrich, 1984; Lanza, 2004)^4^.

Nowadays, though, the most anxiety-inducing form of public transport would appear to be air travel. To give some sense of the problem, 40% of the population in North America experiences some degree of anxiety associated with this form of travel. An estimated 6.5% of them suffer from a fear of flying known as aviophobia (Gould, 2017), while 2.5% have a clinical phobia. This means that they either avoid flying altogether or do so while enduring significant distress (Schaaff, 2019). This is, of course, somewhat ironic given the statistics showing that per mile traveled, one is significantly more likely to die when traveling by road than by air (Schaaff, 2019), as was much commented on following 9/11.

As we will see at several points in this review, driving represents a very particular form of transport inasmuch as there is a potentially important distinction between the driver and any passengers. While both share the same olfactory space/atmosphere, the majority of the multisensory interventions in this area have been targeted at the driver (e.g., alerting/relaxing them) rather than at their passengers^5^. Relevant here, it terms of anxiety, it has been suggested that many motorists appear to drive more dangerously following the introduction of the latest new car safety innovations. The suggestion is that this helps them to maintain a certain desirable level of perceived risk/anxiety, a phenomenon known as “risk compensation” (Peltzman, 1975; Wilde, 1982; Evans and Graham, 1991). Relevant to the theme of the present review, the research shows that ambient scents tend to exert a more positive effect over our mood and well-being when we are stressed/anxious than when we are calm (Warren and Warrenburg, 1993)^6^. Hence, it could be imagined that ambient scent would play a more important role in the context of transportation than in the context of the built environment.

### Personal Space

**Figure 1:**
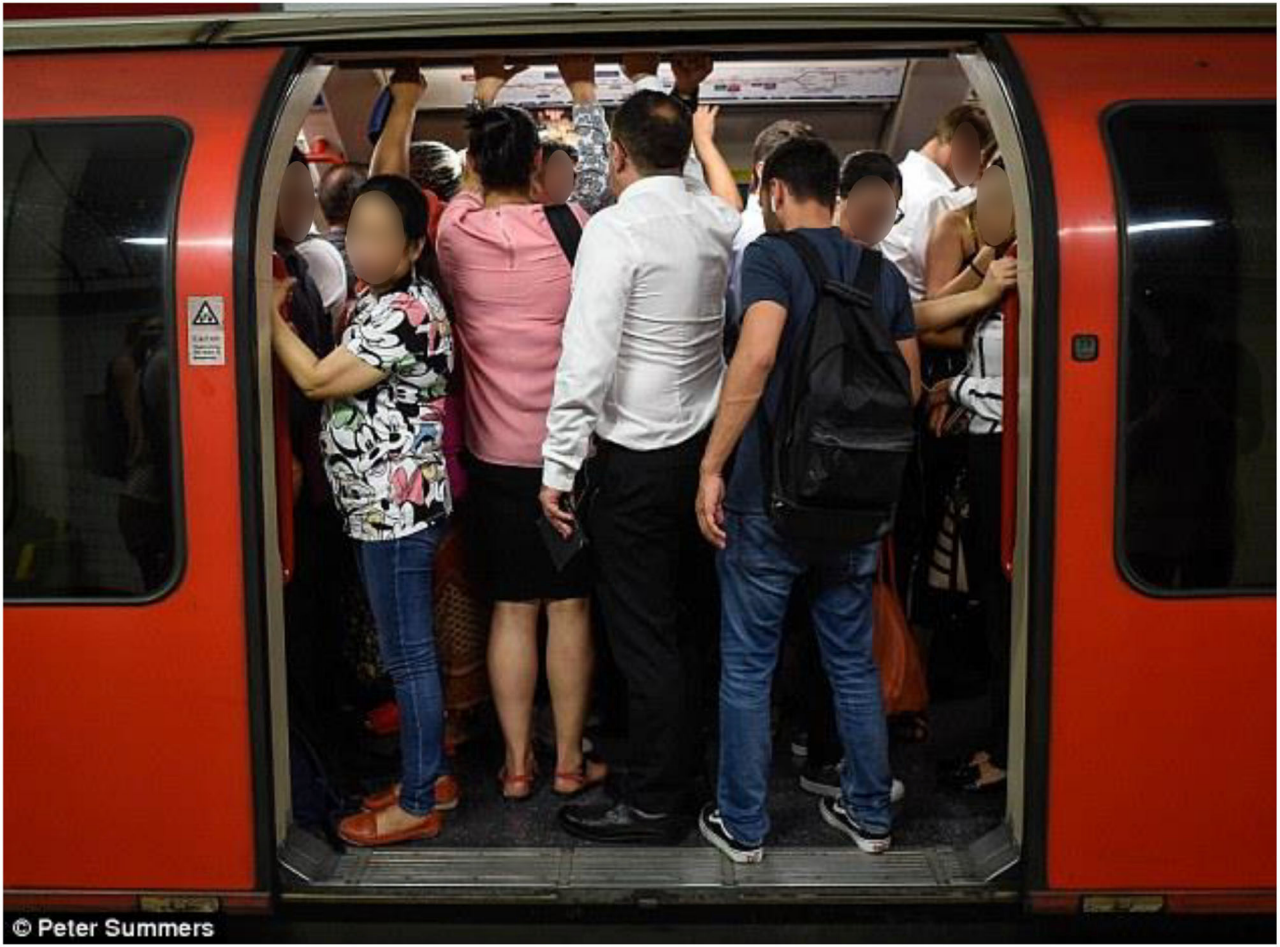
Do we ever get closer to unknown strangers than on the underground? [Copyright P. Summers].

Crowding is also often more likely to be a problem on public transport than is typically the case in the built environment (as highlighted by people's oft-discussed dislike of sitting in the middle seat on the train: McGeeham, 2005; Evans and Wener, 2007)^7^. Interpersonal space is much lower still when it comes to those passengers who are forced to travel in the confined space of mass transportation (be it Mass Transit or the London Tube) at rush hour that pretty much anywhere else (cf. Dipodjoyo, 2019). Is there any other situation, one might ask, where one's personal space is so radically compromised (see Figure 1)? Combined with the often high temperatures (Thompson, 2014)^8^ and lack of natural ventilation, this invasion of the passenger's personal space (Sommer, 1959, 1969; Horowitz et al., 1964; Felipe and Sommer, 1966; Dosey and Meisels, 1969; Kennedy et al., 2009) in the London Tube, but also on other forms of public transport, can soon lead to an awareness of other people's body odors that most of us would rather do without. The overpowering odors can be especially noticeable in the summertime when it can get very hot down there (according to one press report concerning the London Underground, in the summer it is sometimes above the legal temperature for transporting livestock; see Thompson, 2014). The reduced personal space brings the passenger much closer to the smell of unknown others than is typically the case elsewhere in our daily lives. What is more, this is typically found to be an unpleasant experience with those other unknown passengers of other races and/or social classes often suggested to have a distinctive, and frequently disliked, scent (e.g., Orwell, 1937; Largey and Watson, 1972; Classen, 1992; Manalansan, 2006; Bever, 2019)^9^.

One artist who has worked in this space is Angela Ellsworth. In a provocative piece of what I presume can only be called “olfactory performance art,” Ellsworth wore a jersey cocktail dress soaked in her own urine to the opening reception for the Token City installation (a subway simulation) by artist Muriel Magenta at the Arizona State University Art Museum in 1997 (http://museumofwalking.net/works/solo_actual1.html). According to Drobnick (2006), the idea behind the guerrilla art project (going by the name Actual Odor), was precisely to draw people's attention to the way in which malodour destroys any social boundaries that exist in a subway, as it permeates the space and transcends visual barriers or experiences.

### On the Rise of Olfactory Sensitivities

The typically more confined space, and reduced interpersonal distance/space, involved in most forms of public transport differentiates it from our experience of the built environment (Spence, 2020a,b). As we will see below, the confined space may sometimes also increase people's sensitivity to any olfactory manipulation. At the same time, however, it is worth stressing how one of the challenges associated with the deliberate scenting of the built environment in recent decades has been the increasing complaints of those suffering from multiple chemical sensitivity (MCS) syndrome (Lacour et al., 2005). That said, the majority of cases of fragrance sensitivity that have been described in the literature would appear to have been triggered by personal fragrance rather than by ambient scent (Spence, 2002; Byatt, 2003; Fletcher, 2005; Caress and Steinemann, 2009; Steinemann, 2019). In turn, this may perhaps be related to the shift away from the overpoweringly-heavy personal fragrances that were such a distinctive feature of the 1980's, at least in North America)^10^. According to the latest research, such enhanced responsivity to olfactory stimuli appears to reflect a response bias rather than enhanced sensitivity to particular odorants (Andersson et al., 2020). I am not aware of such complaints related to olfactory sensitivities having been tied to the ambient fragrancing of public transport.

## Scent in Motion: The Multiple Roles of Ambient Scent in Different Forms of Transport

Traditional means of getting from a-to-b, such as walking, but also traveling on horseback, bicycle, or motorbike (Pirsig, 1974), or these days, on an e-scooter, have in common exposure to the elements. This typically includes the ambient scent of the built or natural environment through which a person is traveling^11^. What is lost, when this aspect of the experience is denied (e.g., due to anosmia), has been highlighted by various writers (e.g., Tafalla, 2013, 2014). Commentators have also drawn attention to the rich world of scent that pedestrians might encounter when moving through the city via the development of scent maps of cities such as the one made of Amsterdam (e.g., see Degen and Rose, 2012; though see also Tan, 2013; Henshaw, 2014; Poon, 2015; Leimbach, 2017; McLean, 2018). The contemporary experience of scent while passing through the city contrasts radically with the frequent historical complaint concerning the stench of human effluence that nearly always greeted the nostrils of those moving through urban areas in centuries gone by (e.g., Corbin, 1986; Potter, 1999; Jütte, 2005; el-Khoury, 2006; Bradley, 2015; see also Edensor, 2014; Misra, 2015)^12^.

While artists and designers have drawn attention to the olfactory interest/pleasure of a walk through the city, there has been far less research on the impact of such smells, and how they may interact, or be integrated, with other components of the multisensory environment (Ba and Kang, 2019a,b). Though it is worth noting that in these cases the observer is in motion while the scents themselves are static. More often than not, in the examples reviewed below, the scent is delivered from the vehicle or carriage that is itself moving. This observation, once again, hinting at the challenges associated with delivering a clear definition of what constitutes “scent in motion.”

In the rest of this section, I would like to consider various of the major forms of transport^13^, and assess the differing role/opportunity that scent has played. However, rather than organizing this section on the basis of different modes of transport (be it bus, train, tram, or plane, etc.), it seemed more enlightening to organize the review around the four different principal purposes for which scent have seemingly been introduced into passenger transport. These are: masking malodour, olfactory marketing, signature scents, and functional scents.

### Scent in Motion: Masking Malodour/Pollution

As has been noted already, levels of pollution are often much higher in or around transportation than in the built environment. While issues of noise pollution from various means of transport are a major cause for concern (e.g., Burne, 2014; Owen, 2019; Benfield et al., 2020), it is the olfactory, or atmospheric, component of pollution that will be the focus here. That said, it is important to note that many of the pollutants that are so bad for our health are not perceived consciously. One attempt to mask any unpleasant odors in the London Underground took place in 2001, when a pleasant scent by the name of “Madeleine” was introduced for a trial period into several stations. The fragrance had already been introduced successfully into the Paris Metro in 1998, not to mention in the Hong Kong Metro. It must therefore have seemed like a good idea to those in charge of Transport for London to do the same. A scent-encapsulated fragrance described as smelling like “*a fresh, watery floral bouquet of rose and jasmine, combined with citrus top notes, tiny touches of fruit and herbs, giving way to woody accents and a hint of sweetness in the base”* was deposited on the floors in St. James' Park, Euston, and Piccadilly stations to be released when the passengers walked over them.

The confined space on the underground, where people have no choice but to breathe in the air, makes the deliberate introduction of ambient scent a more sensitive and challenging topic than it might be elsewhere. Indeed, the commuters soon started to complain once they became aware of what was going on. In this case, part of the problem, may have been the fragrance itself, which was described by one commuter as a “*cheap fragrance that smells slightly like industrial cleaner. It's a good idea as long as you use a fragrance that doesn't make the station smell like a toilet.”* (quoted in Addley, 2001). It turns out that while the scent released on the various underground systems has the same name, “Madeleine,” its composition is subtly altered depending on the local conditions (Anon, 2001a,b).

Along similar lines, in 2019, a number of the trams on Vienna's U-bahn trialed perfumed carriages following complaints that the underground vehicles were sometimes unpleasantly smelly during summertime (despite widespread air-conditioning). A pleasant yet subtle citrus fruit scent was introduced in order to minimize malodour (Walker, 2019). As is often the case, this olfactory intervention appears to be as much sensory marketing as anything else. One of the other intriguing reasons for bus services and mass transit to introduce a pleasant scent has been in the hope that it might help nudge more people to use public transport (Poon, 2017). In decades gone by, motorists would sometimes hang a scented card (often in the shape of a pine tree or orange) from the rear-view mirror that would slowly release the matching scent thereby hopefully masking any malodour that might otherwise have been perceptible in the car (for a simple sense-hack involving a clothes peg and essential oil, see Cleary, 2021). Even Tesla have come out with a scented card smelling of musk in the shape of Elon Musk's head. According to the website (https://elonsmusk.co/): “*The original Elon Musk air freshener, made with the real scent of Musk. Make your car smell like the world's hardest working billionaire.”* According to press reports, a number of the airlines also pump fragrance into the air conditioning in their planes in order to help eliminate malodour (McGuire, 2017).

### Scent in Motion: Marketing via the Nose

In recent years, a number of more-or-less successful marketing campaigns have been introduced on various forms of public transport in confined spaces such as the Underground. Olfactory marketing has the advantage over other media that in confined spaces such as the Underground, commuters/travelers are exposed to the message no matter where they look or on what they happen to be attending to visually (cf. Nibbe and Orth, 2017). One such olfactory marketing campaign that was introduced into the London Underground two decades ago was pulled after only a day though. The campaign for the Amaretto di Saronno liqueur brand involved releasing the drink's distinctive almond aroma into the underground. The plan had been to release an almond scent into the ventilation system for 2 weeks, hoping to appeal to the olfactory sensibilities of those who found themselves on the tube. Unfortunately, however, the campaign coincided with the release of an article in the country's most widely read newspaper, informing their readers how to recognize signs of terrorist activity. Commuters, especially those on the underground, were warned to be extremely cautious should they detect an almond-like smell, given that the poisonous gas cyanide is made from almonds (albeit bitter almonds), just like the drink (Jury, 2002; Lim, 2014, p. 84)!

**Figure 2:**
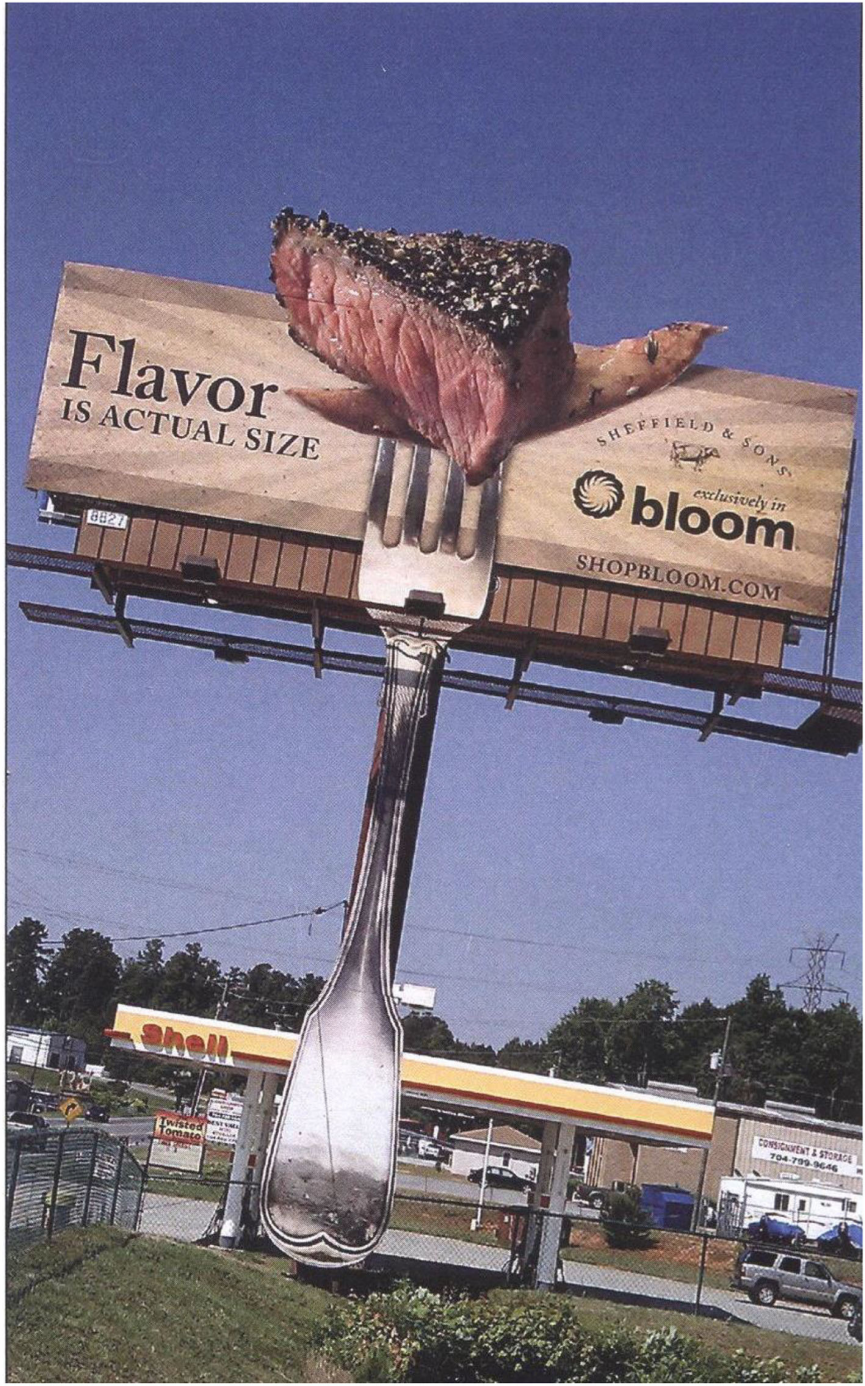
Scented advertisement on Highway 150, North Carolina, USA. In this case, while the commuter is moving, the scent is stationary.

In 2010, on Highway 150 in Moorseville, North Carolina, USA, a huge billboard for Bloom, a division of Food Lion, a grocery store displayed a piece of steak on a fork (see Figure 2). The ScentAir company created a scent that smells of black pepper and BBQ that was pumped out to passing motorists by means of a large fan positioned at its base that blew over a number of BBQ fragrance oil cartridges. The scent was dispersed in the mornings and afternoons when commuter traffic was likely to be at its peak. One bemused motorist described it thus: “*It smells like, uh, barbecue, like hickory or something being barbecued and smells like steak.”* (Aronoff, 2010).

A decade later, scent dispensers were installed on a number of city buses in Seoul, South Korea, that recognized when the Dunkin' Donuts jingle played on the radio. They responded by releasing coffee aroma for the passengers to inhale. The idea behind this Dunkin' Donuts “*Flavor Radio”* campaign was that after stepping off the bus, the passengers would soon enough come across one of the chain's many stores and thus be primed to pay a visit. With a 16% spike in visitors to Dunkin' Donuts branches situated close to a bus stop, not to mention a 29% increase in coffee sales, the evidence suggests that this multisensory marketing strategy (i.e., involving both scent and sound) really did work (Garber, 2012). Others have reported that releasing the smell of coffee on the petrol station forecourt also leads to a dramatic increase in sales (Pape, 2009; Spence, 2015).

**Figure 3:**
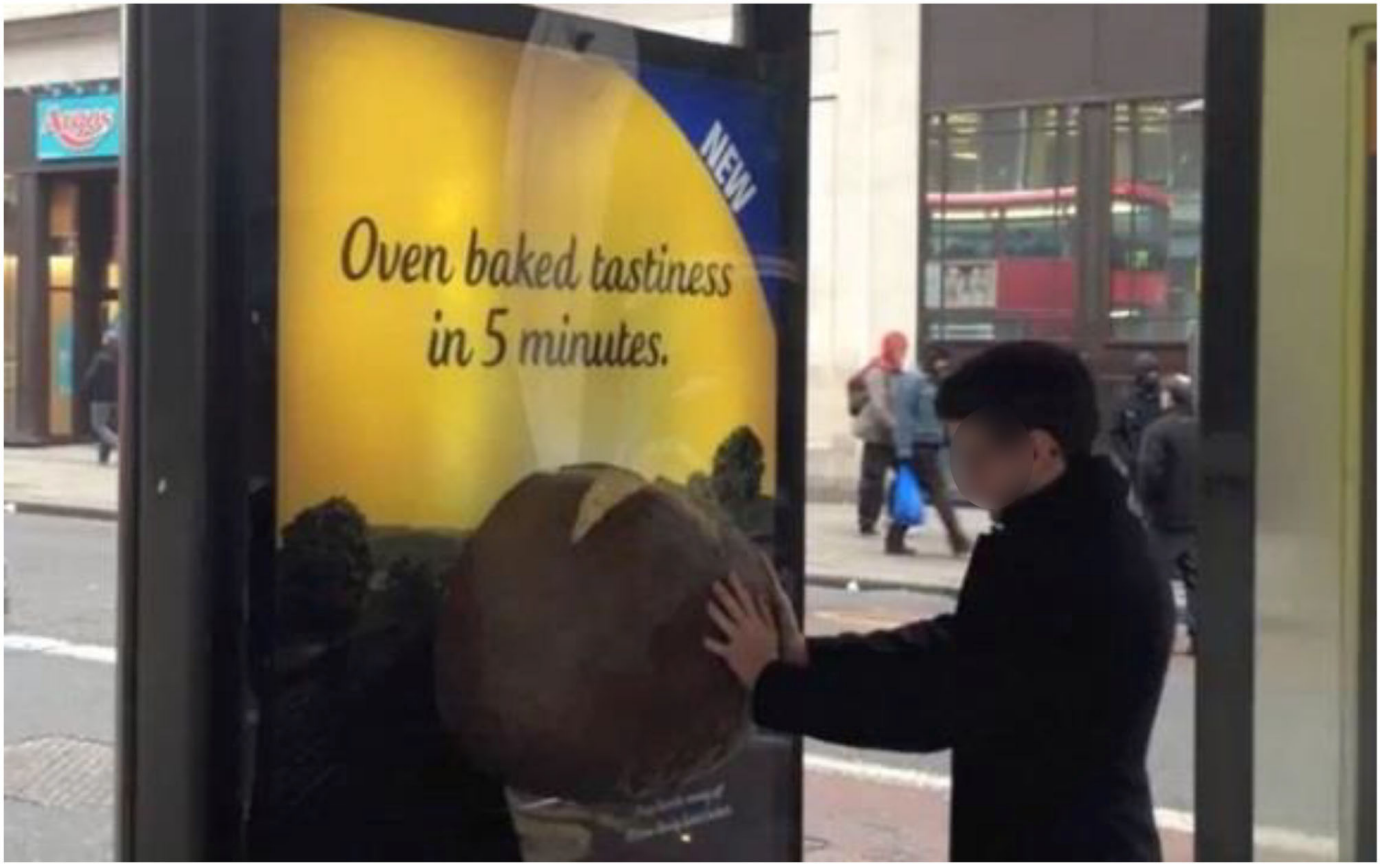
Touch the tattie and smell that delicious oven-baked potato smell. Multisensory marketing at a London bus-stop.

In 2013, I was involved in an olfactory marketing campaign with a London-based marketing agency. The campaign involved a specially-modified London taxi (a black cab) being sent out around the capital's streets pumping-out the smell of McCain's Ready Baked Jackets, basically microwavable oven-baked potatoes (Anon, 2013). At the same time, a few 3D video signs were erected at a number of bus stops. The idea was that curious commuters who chose to push the button on the baked potato were surprised when a pleasant baked potato aroma was released (see Figure 3). In the words of one commentator: “*Each billboard includes a fiberglass potato sculpture and a mysterious button: Push it, and the tuber discharges the aroma of “slow oven-baked jacket potatoes.”*

The following year (i.e., 2014), Tennent Caledonian launched a new drink Lemon T., a light lager mixed with lemon soda. In this case, parts of the Glasgow subway were ambiently-scented with a product-congruent sweet lemon aroma that was supposedly subtle enough not to distract commuters from their journey (Sutton, 2018). Meanwhile, in India, in 2016, the car manufacturer Ford teamed up with advertising agency Kinetic Worldwide to place a number of multisensory billboards to advertise the Ford Mustang at airports (McEleny, 2016). Not only did the motion-activated billboards play the sound of the car's engine to passers-by, but they also emitted smoke to mimic the look and smell of burning tire rubber. Another intriguing example of out-of-home scent marketing comes from Marriott International, who spray scents that are designed to match the destinations offered in its travel program in the vicinity of advertisements displayed in public places (e.g., releasing a coconut aroma for Greece; Mobile Marketing, 2020; Murphy, 2020).

Objections have though, on occasion, been raised to the use of food scents in olfactory marketing. This was exactly what happened when the “Got milk” campaign in California decided that it would be a good idea to start scenting bus shelters with the aroma of cookies in order to give their advertising something of a multisensory boost. The campaign was pulled within days of its launch over concerns that it was grossly-insensitive to the many hungry homeless people in the state who used the shelters to sleep (Cuneo, 2006; Gordon, 2006; Elliott, 2008; Metcalfe, 2012).

What distinguishes the various olfactory marketing campaigns that have been discussed in this section (as well as a couple of other campaigns that took place in British train stations and at Heathrow airport; see Cable, 2014; Polden, 2015) is the typically short-lasting nature of the intervention. These interventions can, though, be seen as part of a wider trend toward the emergence of scent marketing (Morrin, 2010; Sendra-Nadal and Carbonell-Barrachina, 2017; Minsky et al., 2018). As Sutton (2018, p. 132) describes the situation: “*In an industry which has been traditionally dominated by visual stimuli, organizations using Out Of Home (OOH) advertising are increasingly utilizing olfactory elements to entice, engage and entertain target audiences.”* Part of the appeal of scenting public transportation is undoubtedly the captive nature of the audience.

### Signature Scents (Branding)

Given the extensive and growing use of bespoke scent to help deliver a pleasant olfactory identity for everything from sporting venues (Albrecht, 2013; Doll, 2013; Martinez, 2013) to ultra-high-end apartments (Schroeder, 2018)^14^, and from hotel chains (such as the distinctive White Tea scent from the Westin Hotel Group; Kaysen, 2016; Wiedmann et al., 2016; Minsky et al., 2018; Spence, 2018b)^15^ through to retail stores (Spence et al., 2014). In such cases, the fragrance may be blown in through the ductwork, or else delivered by means of stand-alone machines (Kaysen, 2016; Minsky et al., 2018). One challenge, though, is that scent delivered in one room may well end up being distributed through the ductwork to other parts of the building unintentionally contaminating the entire building's ventilation system (see Lai, 2015). One solution, as proposed by Dmitrenko et al. (2017a), is therefore to build a separate air extraction system (i.e., disconnected from the building's central ventilation system) for an indoor space in which a scent is released.

In terms of the signature scent of transportation companies, it has been the airlines who would appear to have led the way. So, for example, Singapore airlines has long used a particular fragrance Stefan Floridian Waters in its planes (Lindstrom, 2005; Strutner, 2015; see also Carey, 2015). Indeed, commentators often comment on how a sense of luxury in the airline sector is perceived in terms of distinctive, bespoke, scent, for instance Singapore Airlines perfume being experienced not just in the cabin but also in the hot towels that passengers are given to refresh themselves (Wiedmann et al., 2016). Looking to the future, premium land-based transportation services might be able to distinguish themselves, and the experience they provide, in part by the use of luxurious signature scent (see https://www.premiumscenting.com/). Hinting at the possibilities in this space, only last year, Ford patented an app to check ride-share cars for bad smells before a passenger gets in (Maddireddy, 2020; Yeung et al., 2020).

Accepting that branded signature scents might be a good idea for the travel industry more generally, the good news is that there are methods available (such as the semantic differential technique, SDT; Dalton et al., 2008) to help align the qualities of the scent with the key brand attributes (cf. Vogt, 2008; Minsky et al., 2018)^16^. It will be an interesting question for future research to determine whether the design of signature scents based on the synaesthetic associations experienced by creative perfumiers, such as Dawn Goldworm, the successful synaesthetic scent director of 12.29, a New York based fragrance design agency (see Kaysen, 2016; Schroeder, 2018) are any more successful than those based on SDT and/or the crossmodal correspondences (see Spence, 2020e, for a review).

### Functional Benefits of Scent in Motion

An extensive body of research shows that ambient scents can affect people's mood, their perception, and also their behavior (Spence, 2020c,d). Perhaps unsurprisingly, therefore, there has been interest in capitalizing on the functional uses of ambient scent in the context of transportation. This has most frequently been discussed/studied in the context of driving where, unlike for most other forms of transport, the person traveling may well also be the one who is in charge of the vehicle. It is worth noting that any attempt to modulate the driver's state of mind (be it counteract sleepiness, or calm the overexcited driver down) via the functional use of scent is inevitably also going to be experienced by any passengers who are traveling (Spence, 2021a)^17^. Ambient in-car fragrance can help to mask malodour and hence potentially (functionally) improve the driver's mood (Ho and Spence, 2008, 2013), and possibly resulting in their driving more safely too (Baron and Kalsher, 1998). Another functional use of scent in the context of driving is to help alert the sleepy motorist (Martin and Cooper, 2007; Raudenbush et al., 2009; Yoshida et al., 2011; Fruhata et al., 2013). At the same time, however, it should be stressed that the higher visual perceptual load of the driver means that their awareness of ambient scent while on the road is likely to be markedly reduced due to what has been termed “inattentional amnesia” (Forster and Spence, 2018).

A number of further functional uses for scent in the context of driving have been articulated recently by the work of Dmitrenko et al. So, for example, in one study, Dmitrenko et al. (2019) examined the use of olfactory notifications to reduce speeding. Meanwhile, the results of a second study highlighted the possibility of using different olfactory cues to notify the driver of different relevant events (Dmitrenko et al., 2017b; see also Hiroike et al., 2009; Okazaki et al., 2018). Finally, these researchers have also reported how olfactory notifications can also be used help to reduce the number of driving-relevant mistakes in the simulator setting (Dmitrenko et al., 2018). Note here also that in 2013 Ford cars applied for a patent concerning a smell notification system for drivers (see Kolich and Ford Global Technologies LLC, 2013). Another intriguing functional use for scent in the context of manual or autonomous driving relates to the alleviation of travel/motion sickness (e.g., Keshavarz et al., 2015; Ranasinghe et al., 2020; Schartmüller and Riener, 2020).

Of course, no one needs a high tech gadget in order to deliver the synthetic scent of nature. As was mentioned a little earlier, drivers have been dangling scent-infused cardboard pine trees from their rear-view mirrors for decades. However, the problem with this solution is that our brains tend to adapt pretty quickly to pleasant or neutral smells. So while you might notice the scent as you open your car door, my guess is that you probably won't think about it much after that. Several studies have demonstrated how the periodic delivery of pulsed scent (e.g., peppermint) can help enhance operator performance (e.g., Warm et al., 1991; Ho and Spence, 2005, 2008; Mahachandra et al., 2015). The Aroma Shooter (http://www.aromajoin.com/) which provides a directional olfactory stream might be especially appropriate for those applications where the scent is targeted specifically at the driver (see also Dmitrenko et al., 2016). However, that said, this particular device is no longer positioned in the vehicular context. There is mention in the US Press of another olfactory delivery device Cyrano being possibly used to deliver scents to drivers (see Baig, 2016).

A few years ago, the British company Jaguar looked into developing a scent display for their vehicles. The idea was for the GPS to periodically check on the car's location and instruct the device to pump the appropriate synthetic natural scent into the cabin. For example, just imagine how much more pleasant it would be to drive through the forest if your nostrils were stimulated by the scent of pine, say, or perhaps the wonderful smell of the earth just after it has rained (geosmin does a pretty good job in this regard). At the same time, however, research from the world of entertainment has highlighted how, unless the scent is chosen very carefully, that ambient pine scent is more likely to remind whoever smells it of a cleaning product, rather than bringing them closer to nature (Spence, 2020d, 2021b), even when the source object (e.g., a pine tree) is clearly visible.

In 2004, Citroen launched their C4 model with a nine-scent olfactory display operating through the ventilation system. The scents were split into groups of three, designed to be congruent with notions of “travel,” “vitality,” and “well-being” and were intended to provide scent for 6 months before it would need to be refilled (Hanlon, 2004). A decade later, Mercedes incorporated an olfactory display in certain of its new models too (Clark, 2013). While such olfactory interventions undoubtedly represent an intriguing direction when it comes to realizing the idea of “scent in motion,” my oft-stated belief is that, as with any other olfactorily-enabled digital device, it will ultimately fail for the simple reason that the customer will not think it worthwhile to buy the refill (Spence et al., 2017).

Of course, cost is likely not to be an issue for the owner of a Bentley car that also offers Sterling silver atomizers to personalize the multisensory experience in their Mulliner edition cars (see https://www.bentleymotors.com/content/brandmaster/master/bentleymotors/en/world-of-bentley/mulliner/personal-commissioning/personalising-your-bentley.html#3f3f8d7bb768277e2e9b127c73364c6f). Meanwhile, BMW have also been working on creating their own in-car perfumes too for their 7-Series (Boeriu, 2015). According to the latter online commentary: “*The optional Ambient Air package features options to ionize the air or fragrance the vehicle interior with selected scents, both of which can be controlled from the air conditioning control console or the iDrive menu. There are three levels of intensity and 8 scents to choose from. The scents can be chosen from the Blue Suite and Green suite, and Golden Suite and Authentic Suite.”*

While it is easy enough to demonstrate the benefits to the user's mood or experience of adding an extra sensory input (Spence, 2021a), the existence of the “fundamental misattribution error” means that as visually-dominant creatures (Hutmacher, 2019), we typically attribute our enhanced pleasure (or experience) to the visual element of a multisensory experience rather than to the olfactory component. The latter, as was mentioned before, may anyway be missed due to the phenomenon of inattentional anosmia (Forster and Spence, 2018). Who knows whether scent displays might 1 day be used to arouse dozy driver instead of the loud and unpleasant auditory alerts that are more commonly used today. Certainly, it is easy to imagine how pumping out an arousing ambient scent such as cinnamon, peppermint, rosemary, eucalyptus, grapefruit (Fruhata et al., 2013) or lemon might prove to be a much less aversive way of achieving the same result than a loud sound, say (Ho and Spence, 2008, 2013). That said, a multisensory approach to modifying the driver's state is likely going to work best here as elsewhere (Bounds, 1996; Ho and Spence, 2008; Spence, 2012; Fruhata et al., 2013).

The functional use of scent has therefore been considered in relation to modifying various aspects of driver performance and experience (Ho and Spence, 2013; Dmitrenko et al., 2016; Mustafa et al., 2016). However, given the rapid adaptation to constant ambient scent, the periodic release of scent holds much more promise as far as the delivery of functional scents is concerned, especially if you want drivers to pay attention to what they are smelling. It would also be good to have more research showing whether sudden-onset olfactory stimuli can automatically (i.e., exogenously) capture attention in a way that constant ambient odors fail to do, often because they are no longer perceived consciously (Funato et al., 2009; Forster and Spence, 2018; though see also Bordegoni et al., 2017; Spence, 2020c).

Another functional use of scent in the context of transportation relates to research showing that those scents we associate with cleanliness can be used to encourage people not to litter/pick up waste (De Lange et al., 2012). So, for example, the presence of an ambient “clean” scent, such as pine or citrus, can help to make a space appear cleaner. The presence of such scents may also help to reduce littering too. At the same time, however, it should be noted that olfactory priming effects have not always proven so easy to replicate (see Smeets and Dijksterhuis, 2014, for a review). There is also a separate line of empirical research, and hence potential opportunity, to use scent functionally to enhance the passengers' multisensory experience/nudge to engage in more prosocial behaviors (e.g., Schiffman and Siebert, 1991; Gueguen, 2001; Spence, 2002, 2021b; Holland et al., 2005; Liljenquist et al., 2010; De Lange et al., 2012; Henshaw et al., 2018), while at the same time possibly also improving their mood (e.g., Warren and Warrenburg, 1993; Spence, 2020c). Finally, it is worth noting how a pleasant scent was introduced onto bus services and mass transit in Singapore the hope that it might help nudge more people to use public transport (cf. Kutzbach, 2010; Poon, 2017). This scent-sory nudging strategy laid bare in the title of a paper in Bloomberg news by Linda Poon (2017): “*To entice riders, Singapore buses get a 'signature scent': Will more people ride public transit if it smells nice?”*

### New Car Smell: A Multifunctional Olfactory Signal

One smell that is often mentioned in the context of transportation and which has been noticeably absent from this review so far is “new car smell” (see Aikman, 1951; Moran, 2000a,b,c). While ratings of which models or marques have the best scent are published annually, suggesting they are distinctive, they have never become differentiated enough to support brand recognition in the absence of other cues^18^. The reason for this is because this evocative smell can be considered as relating to several of the categories outlined above, while not really belonging in any one. Modern car interiors tend to smell terrible unless they are suitably treated (Spence, 2002)^19^. The volatile odors (VOCs) released from the plastic/synthetic materials so often used in car interiors in decades gone by used to make them smell unpleasantly fishy (Shea, 1971; Grabbs et al., 2000; Ritter, 2002). At the same time, however, according to research from California, that oh-so-desirable car smell may actually be carcinogenic (Reddam and Volz, 2021; Watson, 2021).

While many of the car companies have teams of expert noses dedicated to the optimization of the synthetic smell that greets the customer (see Moran, 2000b), these scents do not really qualify as signature scents for a couple of reasons: On the one hand, the consumer considers it the smell of the vehicle (i.e., product-intrinsic; Van Lente and Herman, 2001), rather than of the brand. On the other, while different marques do smell somewhat different (i.e., some new cars do smell better than others), the relevant question is whether drivers are able to distinguish between the smell of a new Volkswagen (VW) vs. Ford car, say. In the absence of evidence, my guess is that this distinction is simply too subtle for most drivers to be able to recognize the marque reliably solely on the basis of the new car smell. In fact, it is an intriguing question is to just how many high street brands actually have a distinctive, and instantly recognizable, signature scent beyond the likes of Lush, Subway, Cinnabon, and perhaps a few others (see Nassauer, 2014). One can only wonder how distinctive the signature scent, Nuance, introduced by General Motors for its Cadillac car in 2003 may have been (Lindstrom, 2005).

SC Gordon Ltd., the coachbuilders of Rolls-Royce cars, have developed their own unique new car smell designed specifically to mimic the aromatic blend of leather and wood of a vintage 1965 Silver Cloud model! The car cologne is applied when new cars come in for repair, and according to Hugh Hadland, Managing Director of the company, “*People say they don't understand what we've done, but that their cars come back different and better.”* It seems that just one dash of the luxury perfume is enough to restore that sense of luxury in even the most expensive of consumer purchases (Seat sniffers, 2000), and can even be used to add value when people come to resell their own car (Aikman, 1951; Hamilton, 1966; Wright, 1966). Intriguingly, surveys consistently highlight a strong relationship between how much consumers like a vehicle's interior smell and how they rate the vehicle's interior overall (Power and Associates, 2000). One other intriguing question here concerns the role of new car scent in the seemingly irrational desire to buy new car—-given how rapidly they are known to depreciate (Rohrer, 2008). Intriguingly, new car smell is not appreciated by everyone. Truong (2018) has reported that: “According to JD Power, more than 10% of drivers in China—the world's largest auto market—complained about the new-car smell in its 2018 survey.” Such findings have resulted in Ford filing a patent for a technique to strip its new models of their new car smell in the region.

### Food Scents in Transportation

Before moving on, it is perhaps worth noting in passing that food and drink are often consumed while travelers are on the move. For instance according to an interview with Michael Pollen, North Americans consume 20% of their meals while at the wheel in their cars (https://news.stanford.edu/news/multi/features/food/eating.html). This is what is sometimes referred to as dashboard dining or cup-holder cuisine (Hill, 2005; see also Morrison, 2021, for one view of the future of dining in autonomous cars). Designed food experience for passengers on other forms of transport has also occupied the minds of many researchers' (e.g., Horwitz and Singley, 2004; Muecke, 2004; Spence, 2017a,b; Taylor et al., 2019, 2020). Returning to a theme that was mentioned earlier in this review, it has been suggested by de Syon (2008) that the provision of food on airplanes may also be one means of helping passengers to manage their anxiety (though more could certainly be done in this regard; e.g., Delahaye, 2017).

Much of the profit, at least amongst the budget airlines comes from the sale of food and drink, and the on-board duty free shop (Ciesluk, 2020). As such, this must raise the temptation to use smell to sell. Similarly, think only of how the food and beverage offering is used as one of the key differentiators between different classes of service in the air (Economy, Business, First; O'Flaherty, 2015; Spence, 2017a,b). British trains, with their distinction between Standard and First Class carriages, also use the food offering to discriminate between the different classes of service. The challenge in certain transportation situations is to deal with the deleterious effects of the dry air/lowered cabin air pressure, not to mention the 80 dB of background noise, as has been documented in the case of delivering tasty food in the air (Spence, 2017a,b,c; Spence, 2018a).

As such, one probably needs to consider how the smell associated with any F&B offering may influence the olfactory atmosphere, not only for those who are eating, but also for those who are not. Indeed, consuming certain forms of food are banned on public transport in some transport systems. For example, it is forbidden to drink alcohol or eat hot foods on many forms of public transport in the UK and elsewhere (see Hello Magazine, 2017). The latter prohibition presumably designed to minimize the olfactory discomfort to the other passengers), while chewing gum has long been banned on public transit in Singapore (presumably to help reduce litter and mess; Metz, 2015). One example of what goes wrong if one doesn't take account of what the aroma of food may remind the passenger of is highlighted by the case of Virgin Australia. The smell of parmesan sandwiches were misinterpreted as the smell of sweaty old socks (both share the volatile compound valeric acid), and made many of the passengers fall sick (Buaya, 2016)^20^.

### Future Travel: Space Tourism

There is growing excitement about the impending emergence of space tourism (Henderson and Tsui, 2019). As several different companies (SpaceX, Virgin Galactic, Space Blue Origin, etc.) compete to be the first to offer a commercial service, important questions remain about the nature of the multisensory experience that the customers will be offered (Obrist et al., 2019). The starting point is not good, given that reports from astronauts describing space as smelling like: “*gunpowder, hot metal, welding”* (New York Hall of Science, 2016). The repeated recycling of limited air can potentially lead to the build-up of volatile pollutants (Taylor et al., 2019, 2020). At the same time, however, the lowered gravitational pull also leads to the build up of blood in the head, and this may constrict the nasal airways (NPR, 2012), leading to what is known as “space anosmia” (Varma et al., 2000). However, a more pressing problem might be to deal with space sickness that many feel on entering space (Crampton, 1993). As such, any scent that can help to reduce anxiety and/or counteract the effect of travel sickness might be considered a good idea (though see Paillard et al., 2014; Keshavarz et al., 2015).

## Scented Travel: Scented Terminals and Stations

Commercial travel often involves passengers transiting through terminals both prior to, and more briefly upon, arrival. Think of train stations, airports, subway/metro stations, and even bus/tram stops. Such spaces constitute an integral part of the experience of travel. In fact, looking back the discussion of scenting the metro/underground involved the smell of the terminal not the transportation itself (i.e., the carriages). By contrast, when discussing the scent of air travel, the focus was very much on the scent of the airplanes/airlines rather than the airports (see Klara, 2012). That said, there has been interest in scenting the airline passenger's experience prior to take-off. For instance, two decades ago, British Airways (BA) introduced a functional scent into their airport lounges (Spence, 2002). According to a report that appeared in *The Wall Street Journal* (Ellison and White, 2000), a signature scent called “Meadow Grass” was introduced into their executive airport lounges. The idea was that the weary business traveler would be greeted by a familiar scent as soon as they walked into the lounge (no matter what continent they happened to be on), signaling that their journey was near its end (or presumably, just beginning). It is noticeable how contemporary scenting strategies tend to try and ensure a consistent olfactory identity across all touch points (i.e., including both airline lounges and the airplane itself). For instance, Delta Airlines deliberately used a fragrance of orange peel, sandalwood, cedar and leather, part of a move to create a signature airline aroma to charm passengers in airport lounge lobbies, such as at Chicago's O'Hare airport (Carey, 2015).

The olfactory ambience of airports would seem to differ substantially as a function of the country that one happens to be transiting through. While most airports are olfactorily neutral^21^, the atmosphere in North American airports tends to be heavy with the smell of food franchises (see Nassauer, 2014). The broadly appealing smell of coffee has become a distinctive smell of train stations and many other public spaces. And while there has been some discussion of given entire shopping centers a branded scent (see Spence et al., 2014), I haven't heard anything similar for airports as yet. That said, pre-Covid-19, I was always struck flying in to Heathrow by the cheap clean fragrance, my response then much like that of those quoted earlier regarding the scenting of the underground that was disbursed throughout the terminal—-from what source I never was able to ascertain. In this case, it is unclear into which category we should put the scenting strategy.

Intriguingly, there are also examples of olfactory performance artists who have worked with the scent of such familiar, yet often transitorily experienced, spaces. Consider here only the work of Helgard Haug, a young performance artist who won a prize in support of a public art piece at the subway station Berlin Alexanderplatz, once the social center of East Berlin. In 2000, Haug commissioned a distillation of the scents of Berlin Alexanderplatz that were presented in tiny souvenir glass vials dispensed in the station. The perfumer designed the scent based on his own perception of the station without chemical analysis. U-deur included the smell of bread as one of the primary odors (because there was once a bakery stand in the subway) along with the smells of cleaning agents, oil, and electricity. According to Drobnick (2006), the public response to the project was apparently extraordinary. People wrote that the little sniff-bottle brought to mind memories and associations with the smells of a divided Berlin, for instance, the “dead” stations that West Berlin subway trains went through after passing the Wall. At the same time, thoughts about the Stasi archive with its items saturated with the body odor of East German criminals and dissidents were also triggered in the minds of some.

## Challenges With Controlling the Olfactory Environment

The technical means of introducing and controlling scent, and, more importantly, getting the level right, is by no easy, especially in the case of scent in motion. What is more, olfactory adaptation/habituation means that those working in a scented environment may soon lose any awareness of the scent whereas occasional passengers may find the scent to be much stronger (Spence, 2020c). Furthermore, too often (or so it would seem), cheap synthetic scents (or at least scents that are perceived as such) are used. Creating a bespoke solutions can be expensive (Spence et al., 2017), and trade marking specific scents is a challenging business (Hammersley, 1998) thus making innovation in this space even more challenging.

Most successful approaches to scenting the environment, be it in the context of scenting the built environment or scent in motion, as discussed here, do not occur in isolation, but rather involve the coordinated stimulation of multiple senses, be it in the car (e.g., Bijsterveld et al., 2014), in the underground/underground car-park or metro station (Sayin et al., 2015), or while in the air. Here it is important to stress that multisensory interactions often influence olfactory perception (e.g., Velasco et al., 2014). As such, it can be hard to know how exactly a scent will be perceived until it is actually introduced *in situ*. Indeed, problems associated with sensory overload and sensory incongruency (Malhotra, 1984; Spence, 2020a,b) have been used to explain why the benefits of adding one sense sometimes disappear in real-world interventions when scent and sound interventions have been combined (e.g., Mattila and Wirtz, 2001; Morrin and Chebat, 2005; Fenko and Loock, 2014; see also Spangenberg et al., 2005).

## Conclusions

While those thinking about the scent of transport in the modern era have often focused on masking the scent of the typically confined space, or of the passengers – both those who are currently present (Walker, 2019) and those who have long since left their olfactory mark (see Robinson, 2016; see also Anon, 2015)—there is also growing interest in branding the experience by means of the introduction of signature scents. To date, this has primarily occurred in the context of the airlines (Lindstrom, 2005; Carey, 2015; Strutner, 2015; Wiedmann et al., 2016), but there seems little reason to believe that the approach will not be extended to other forms of transportation, especially given the dynamic nature of the sector currently (cf. Baskas, 2019). It is crucial, especially at the luxury end of the market, that signature scents are used as part of a multisensory marketing/design strategy (Wiedmann et al., 2016; Spence, 2021a). Typically, the most effective sensory interventions engage multiple senses (see Bounds, 1996; Spence, 2012; Fruhata et al., 2013), while ensuring congruency across multiple sensory touchpoints and avoiding the dangers of sensory overload (Spence, 2021a).

At present, the smell is just one of the aspects of the experience of luxury travel that those tempting their customers with the virtual reality (VR) experience before they travel typically fails to capture (Yerman, 2015; though see also Cable, 2014, for the use of scent to help mentally transport travelers to their destination; and Flavián et al., 2021, for the latest research in this area). Perhaps also worth mentioning here are the various that olfactory-displays “on the go” that have been enabled by head-mounted displays (HMDs; e.g., Yanagida et al., 2004; Howell et al., 2016; Ranasinghe et al., 2018; Comşa et al., 2019; Saleme et al., 2019; Nakamoto et al., 2020).

A number of scent-based marketing interventions, although typically short-lived, have also been associated with the theme of scent in motion in recent years (although in such cases it is more often the case that the scent itself is static, while the traveler/passenger passes through, or by the scented location). Scent-based marketing interventions, although not yet widespread, continue to flourish (e.g., Garber, 2012; Anon, 2013; Cable, 2014; Polden, 2015; McEleny, 2016; Mobile Marketing, 2020; Murphy, 2020). That said, as we have seen here, problems have, on occasion, arisen in part due to confined space and the involuntary nature of the passenger's engagement with the olfactory stimulus, especially in confined space (cf. Anon, 2001a,b; Jury, 2002; Lim, 2014). Looking to the future, it would seem likely that the development of functional branded scents will increasingly be a feature of our experience of travel (Ho and Spence, 2008, 2013; Spence, 2021a), at the interface between the more artistic and scientific worlds of fragrance (Drobnick, 2006).

The functional use of scent in a passenger transport/travel context includes everything from the suggestion that ambient scents could be used to help reduce anxiety amongst the 40% of passengers who report that flying makes them anxious (Strutner, 2015) through to attempts to use pleasant scent in order to help tackle motion sickness (Keshavarz et al., 2015; Ranasinghe et al., 2020; Schartmüller and Riener, 2020). Some are even considering whether scent can be used strategically to help nudge more people to take more sustainable and environmentally friendly forms of transport (Poon, 2017). After all, it is worth noting that travel is typically involved when we go on holiday, with the global tourism market estimated to be worth a trillion dollars annually (World Travel Tourism Council, 2017). As such, anything that can be done to help reduce global tourism's carbon footprint, estimated in 2018 (i.e., pre-Covid-19) to represent 8% of global greenhouse gas emissions (Lenzen et al., 2018), through sensory nudging (possibly involving olfaction) toward more environmentally-friendly forms of transportation, would seem like an avenue that has to be worth pursuing in the future (cf. Poon, 2017).

One area of particular interest regarding the future of olfactory displays in the automative industry, relates to the emerging benefits of the nature effect (Williams, 2017; Spence, 2021a), the olfactory version of which one might call aromatherapy (Spence, 2003). It will be interesting to see whether the benefits of the nature effect can be incorporated thus delivering a benefit for driver's well-being. It is also interesting here to note how some car companies have been trying to develop vehicle interiors that are so pleasurable that people choose to sit in their car even once their journey has ended. It would seem that getting the olfactory atmosphere right will be a key component to the success of any such enterprise. Another suggestion here relates to the emerging problem about how to facilitate switching between being a passive passenger and an active driver semi-autonomous vehicles. Olfactory signaling might help to mark out these different states with driving presumably being associated with a more alerting/arousing state of mind, while the other is presumably more relaxing. Finally here, one might also wonder whether scents could potentially be used to help wayfinding while driving (cf. Hamburger and Knauff, 2019).

Finally, there is a sense in which, at least to your present author, when the proposed form of transport being discussed, moves further into the future, such as for example, Virgin Hyperloop (Giacobbe, 2021), that realizing what taking this new form of transport will smell like somehow makes it more believable, “more real.” One might argue that scent, while an important part of the experience of any form of transportation, would be figured out later in the day (given its aesthetic rather than functional role). That scent design (along with sound design) is brought forward in the design process, etc. seems to be playing a role, perhaps a fifth role of olfaction, in making one hypothetical version of the future of transportation, of scent in motion, more believable, somehow more tangible.

## Author Contributions

CS wrote and researched all parts of this review.

## Conflict of Interest

The author declares that the research was conducted in the absence of any commercial or financial relationships that could be construed as a potential conflict of interest.

## Footnotes

^1^Currently, around 55% of the population live are thought to live in urban areas, up from 30% in 1950, see https://www.un.org/development/desa/en/news/population/2018-revision-of-world-urbanization-prospects.html.

^2^Though, on reflection, perhaps this isn't all that surprising given that the more pollution there is, the more cars one might expect there to be on the roads, all else being equal.

^3^That said, the incidence of SBS (Love, 2018) would appear to be in decline, perhaps due to improved ventilation.

^4^Relevant to the present review, while “elevator music” became an expression in its own right, I have heard no mention of elevator scent (though see Robinson, 2016; Valenti, 2018, for the Elevator Scent launched by Off White and Byredo). One concern, though, about introducing scent in this case is the highly-confined nature of the space.

^5^The rise of the semi-autonomous and fully autonomous vehicles is, though, slowly starting to blur this particular distinction (Anon, 2016; Kurczewski, 2020). Relevant here, the potential use of scent to enhance an individual's trust in the context of autonomous driving represents a particularly intriguing direction for research (see Wintersberger et al., 2019).

^6^After a decade of research studying the impact of fragrance on mood and well-being for International Flavors & Fragrances (IFF), one of the world's largest flavor houses, Warren and Warrenburg (1993, p. 9) arrived at the following conclusions: “*1. Fragrance-evoked mood changes are small, but beneficial to our well-being. 2. Fragrance can be used to reduce the stress response in humans, but its physiological effects on a non-stressed subject are minimal and difficult to measure.”*.

^7^Meanwhile, keeping the middle seat on the airplane empty has been suggested to be an effective strategy to reduce Covid-19 infection rates while flying (Matthews, 2020).

^8^Note that high temperatures have also been linked to increased anti-social behavior (Baron and Ransberger, 1978).

^9^This, one of the themes brought out in Oscar-winning Korean movie, Parasite – a very dark comedy indeed (Lawless, 2020)!.

^10^As an article in The New York Times in 1988 put it: “*The American woman has acquired a taste for eaux de toilettes and colognes that are unusually strong and lasting”* (Wells, 1988).

^11^Along these lines, one of the protagonists, Martha Coffin Wright in Dorothy Wickenden's The agitators, writes in a letter about the members of the pubic watching a wedding in 1860 in the United States that they were there: “to hear the music, and ‘take a smell” (Wickenden, 2021, p. 162).

^12^Intriguingly, though, some Ancient Roman sources actually have the city-dwellers complaining about the foul goaty/garlicky smell that greeted their nostrils on visiting the countryside (Morley, 2015, p. 117).

^13^Transport is defined as taking or carrying (people or goods) from one place to another by means of a vehicle, aircraft, or ship etc.

^14^It should though, be noted that some commentators believe that the scenting of different spaces in new building developments is nothing more than a gimmick (Kaysen, 2016).

^15^Perhaps triggered by claims that people will refuse to check in if they detect a strange smell in the lobby of a hotel (Pacelle, 1992).

^16^Though here it should also be noted that cultural (not to mention individual) differences in the meaning/association of scents have been reported (Trivedi, 2006; see also Truong, 2018).

^17^Of course, this concern also applies to the use of auditory alerts too (see Ho and Spence, 2008).

^18^Luxury yachts represent a vibrant niche market (one might consider, new yacht smell for the superrich?; https://yachtscent.com/), and holiday cruises are very big business, representing the fastest growing sector of the travel market, at least they were pre-Covid-19 (Giese, 2020). However, I am not aware of any research on the role of scent in this particular environment (though see Hood, 2019, for the recent introduction of a signature fragrance, Ship No. 1, linked to Virgin Voyages cruises).

^19^Some decades ago now, Rolls-Royce even ran a scented add in Architectural Digest that allowed readers to smell the leathery Rolls interior (Gibbs, 1988).

^20^Here one is reminded of Mark Twain's (Clemens, 1957) “The Invalid's Story” in which a man becomes increasingly distressed while riding in a freight car with what he imagines can only be a coffin containing a pungent, putrid corpse. Unable to tolerate the nauseating smell any longer, he eventually jumps off the train into a winter storm, contracting a fatal illness. However, before he dies, he learns that the coffin in the freight car was actually a large crate of cheese.

^21^And of course, the duty free fragrance counters are a distinctive feature of the majority of airports, though the scent is typically not widely dispersed.
